# c-MYC Triggers Lipid Remodelling During Early Somatic Cell Reprogramming to Pluripotency

**DOI:** 10.1007/s12015-021-10239-2

**Published:** 2021-09-02

**Authors:** Javier Prieto, Juan Carlos García-Cañaveras, Marian León, Ramón Sendra, Xavier Ponsoda, Juan Carlos Izpisúa Belmonte, Agustín Lahoz, Josema Torres

**Affiliations:** 1 Departamento Biología Celular, Biología Funcional y Antropología Física, Universitat de València, 46100 Burjassot, Valencia Spain; 2 Gene Expression Laboratory, Salk Institute for Biological Studies, La Jolla, CA 92037 USA; 3 Biomarkers and Precision Medicine Unit, Instituto de Investigación Sanitaria La Fe, 46026 Valencia, Spain; 4 Departamento de Bioquímica y Biología Molecular, Universitat de València, 46100 Burjassot, Valencia Spain; 5 Instituto de Investigación Sanitaria (INCLIVA), 46010 Valencia, Spain

## Abstract

Metabolic rewiring and mitochondrial dynamics remodelling are hallmarks of cell reprogramming, but the roles of the reprogramming factors in these changes are not fully understood. Here we show that c-MYC induces biosynthesis of fatty acids and increases the rate of pentose phosphate pathway. Time-course profiling of fatty acids and complex lipids during cell reprogramming using lipidomics revealed a profound remodelling of the lipid content, as well as the saturation and length of their acyl chains, in a c-MYC-dependent manner. Pluripotent cells displayed abundant cardiolipins and scarce phosphatidylcholines, with a prevalence of monounsaturated acyl chains. Cells undergoing cell reprogramming showed an increase in mitochondrial membrane potential that paralleled that of mitochondrial-specific cardiolipins. We conclude that c-MYC controls the rewiring of somatic cell metabolism early in cell reprogramming by orchestrating cell proliferation, synthesis of macromolecular components and lipid remodelling, all necessary processes for a successful phenotypic transition to pluripotency.

**Graphical Abstract:**

c-MYC promotes anabolic metabolism, mitochondrial fitness and lipid remodelling early in cell reprogramming.

A high rate of aerobic glycolysis is crucial to provide intermediaries for biosynthetic pathways. To ensure the availability of nucleotides, amino acids and lipids for cell proliferation, cells must provide with a constant flux of the elemental building blocks for macromolecule assembly and fulfil the anabolic demands to reach the critical cellular mass levels to satisfactorily undergo cell division.

A high rate of aerobic glycolysis is induced by c-MYC, increasing the amounts of intracellular Glucose-6-phosphate (G6P), fructose-6-phosphate (F6P), and glyceraldehyde-3-phosphate (GA3P), which can all enter pentose phosphate pathway (PPP) to produce Ribose-5-Phosphate (R5P) and NADPH, which are necessary for the biosynthesis of biomolecules such as proteins, nucleic acids, or lipids. C-MYC-dependent activation of glucose-6-phosphate dehydrogenase (G6PD) may play a critical role in the shunting of G6P to PPP and generation of NADPH. High glycolytic flux increases the amounts of dihydroxyacetone phosphate (DHAP), which is crucial for biosynthesis of phospholipids and triacylglycerols, and pyruvate (Pyr), which can be converted to citrate (Cit) in the mitochondria and enter the biosynthesis of fatty acids (FA). During cell reprogramming, c-MYC-dependent lipid remodelling leads to Polyunsaturated Fatty Acid (PUFA) downregulation and Monounsaturated Fatty Acid (MUFA) upregulation, which may play critical roles in cytoarchitectural remodelling of cell membrane or non-canonical autophagy, respectively. Cardiolipin (pink dots) rise early in cell reprogramming correlates with an increase in mitochondrial fitness, suggesting that c-MYC may restore proper levels of cardiolipins and antioxidant proteins, such as UCP2, to guarantee an optimal mitochondrial function while upholding ROS levels, reinforcing the idea of cell rejuvenation early in cell reprogramming.

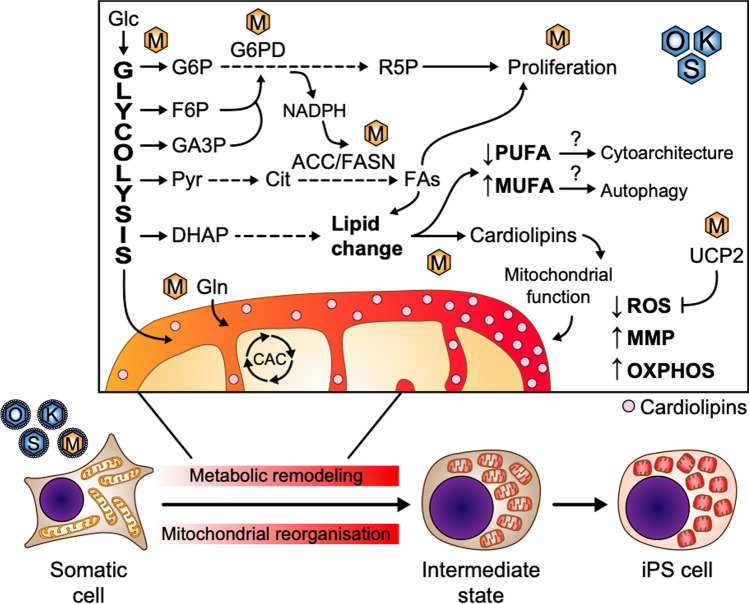

## Introduction

Somatic cells can be reprogrammed to a pluripotent state similar to that of embryonic stem cells (ESCs) [1]. Among the different approaches to achieve cell reprogramming, ectopic expression of OCT4, SOX2, KLF4, and c-MYC using retroviruses that encode for these four factors (OSKM hereinafter) has become the most widespread technique to obtain induced pluripotent stem cells (iPSCs) due to its high reproducibility, applicability to human samples and simplicity of the process.

There are important differences between somatic and pluripotent stem cells (PSCs), consequently this transformation entails a deep reorganization of the cellular phenotype at all levels. This dramatic phenotypic transformation requires an organized sequence of events to be concluded successfully. In this regard, genome-wide and proteomic studies have shown that cell reprogramming is a multi-step process organized in two waves or cascades of cellular and molecular processes [2]. The first wave or stochastic phase is fundamentally associated with changes in the cell cycle, metabolism, and cytoarchitecture. As a result, a small subset of cells can reach the second wave or deterministic phase, where cells undergo a reactivation of the endogenous core pluripotency network, which controls pluripotency independently of the exogenous factors or stimuli used for cell reprogramming.

Mitochondrial network and ultrastructure reorganization, together with a switch in metabolism both to obtain energy and deliver metabolites for increasing cell mass, are hallmarks of cell reprogramming [3]. During the stochastic phase, somatic cells transit from an oxidative phosphorylation (OXPHOS)-based metabolism, with elongated and cristae-rich mitochondria, to a metabolic state substantiated on aerobic glycolysis, with fragmented and cristae-poor mitochondria [4]. Previously, we and others have shown that c-MYC plays a central role in modulating these metabolic pathways and inducing mitochondrial fission to favour somatic cell fate change to iPSCs [5, 6]. c-MYC is a well-known proto-oncogene and there is a clear parallel between c-MYC expression and cell proliferation [7]. As the activation of cell cycle is an imperative first step towards cell reprogramming [8], it is not devious to suggest that c-MYC is an essential factor in cell reprogramming by inducing cell cycle entry while orchestrating both mitochondrial dynamics and metabolic changes to properly pave the way to pluripotency.

Under conditions of high cell proliferation, the Warburg effect is essential to acquire the optimal metabolic context to provide enough substrates to guarantee biosynthesis of the macromolecular components, such as nucleic acids, proteins, and lipids, all necessary for increasing cell mass [9]. In this regard, ESCs [10, 11] and neural stem cells (NSCs) [12] have a high expression of the genes encoding for the enzymes ATP-citrate lyase (*Acly*), Acetyl-CoA carboxylase (*Acc*) and fatty acid synthase (*Fasn*), three crucial enzymes in the biosynthesis of fatty acids. The absence of *Fasn* in mouse NSCs impairs neurogenesis and proliferation of neural progenitors [12] and the chemical inhibition of ACC and FASN impairs acquisition of pluripotency during cell reprogramming [11]. In parallel, the silencing or inhibition of ACLY [13, 14] ACC [15, 16] or FASN [17, 18] dramatically reduces the proliferation and survival of tumor cells *in vitro* and the role of pentose phosphate pathway has been well-described in cancer [19].

Both pathways, biosynthesis of fatty acids and pentose phosphate pathway, are necessary for lipid remodeling, providing acyl chains and NAPDH, respectively [20]. Lipids play a wide variety of key roles in the cell, such as membrane components [21], energy and heat sources [22], signalling molecules [23] and substrates for protein modification [24]. Underpinning all these functions there is a wide diversity of lipids. Attending to the chemical nature and partner interactions, there are several examples of well-described effects of lipids on important cellular processes, such as 1) fission (phosphatidylethanolamine) [25] and fusion (lyso-phosphatidic acid) of organelles [26], 2) thickness, fluidity (cholesterol and sphingolipids) [27] and bending rigidity (polyunsaturated fatty acids) [28, 29] of biological membranes, 3) vesicular trafficking through ‘packing defect’ (polyunsaturated and monounsaturated fatty acids) [30], 4) protein-to-membrane recruitment (phosphoinositide, phosphatidic acid, diacylglycerol and phosphatidylserine) [31], 5) mitochondrial dynamics and performance (cardiolipins) [32] or 6) apoptosis (cardiolipins) [32].

It is known that c-MYC favours the biosynthesis of nucleic acids and lipids in cancer and somatic cells [4]. However, the role of this protooncogene in synchronising the activation of the afore mentioned biosynthetic pathways with mitochondrial fitness during early cell reprogramming has not been investigated. We therefore sought to analyse the role of c-MYC in orchestrating the activation of anabolic metabolism and energy production to safely boost the progression of somatic cells through the early stages of cell reprogramming.

## Materials and Methods

### Cell Culture, Reprogramming Assays, Reagents and Plasmids

E14Tg2a mouse ESCs, a gift of Prof. Austin G. Smith, were cultured on gelatinised plates in ESC medium supplemented with 10% FBS (Hyclone) in the presence of LIF. The iPSC lines used in this study have been described elsewhere [33, 34]. When indicated, ESCs or iPSCs were grown on gelatinised plates in 2i medium (a mixture (1:1) of Neurobasal (ThermoFisher Scientific) and DMEMF12 (Biowest), supplemented with 0.5 × N2 (ThermoFisher Scientific), 0.5 × B27 (Fisher Scientific), 3 μM CHIR99021 (Merck) and 1 μM PD0325901 (Merck)) in the presence of hLIF (made in house) [35]. PlatE and SNL cells were grown in high glucose DMEM (Biowest) containing 10% FBS. When indicated, SNL cells were mitotically inactivated by treatment with 4 μg/ml Mitomycin-C (Merck) for 2.5 h at 37 °C. Wild type mouse embryonic fibroblasts (MEFs) (homogenous C57BL/6 background) were prepared from E13.5 pooled embryos and cultured in high glucose DMEM supplemented with 10% FBS and 1 × Penicillin/Streptomycin (ThermoFisher Scientific). The retroviral vectors pMX-*Oct4*, pMX-*Sox2*, pMX-*Klf4*, and pMX-c-*Myc* were from Addgene [1].

Reprogramming was carried out by transduction of MEFs with retroviruses encoding OCT4, SOX2, KLF4 and c-MYC (referred in the text as OSKM) or OCT4, SOX2 and KLF4 (referred in the text as OSK) as previously described [33, 34]. Ecotropic retroviruses were produced in PlatE cells transfected using Polyethylenimine (PEI) “Max” (Mw 40 000) (Polysciences) exactly as described [6]. For reprogramming, 8 × 10^5^ MEFs were plated per p100 mm the day before the assay. Next day (day 0), MEFs were incubated overnight with a 1:1:1:1 mixture of mouse *Oct4*, *Sox2*, *Klf4*, and *c-Myc* retroviral supernatants supplemented with 4 μg/ml Polybrene (Merck). The next day, the supernatants were replaced with fresh media, and cells were incubated for 3 more days (day 4). Then, 5 × 10^4^ cells were plated on a confluent layer of mitotically inactivated SNL feeders seeded the day before on gelatin-coated p60 mm at 2.5 × 10^6^ cells per dish. The next day (day5), media was changed to ESC growth media containing 15% FBS and hLIF. The media was changed every other day. When indicated, cell reprogramming was conducted in the presence of DMSO (Merck) as vehicle control, 100 μM of G6PD inhibitor DHEA (Selleckchem) or 200 μM of the FASN inhibitor C75 (Tocris Bioscience). Reprogramming was assessed 25 days after transduction of MEFs with OSKM-encoding retroviruses by scoring all the alkaline phosphatase positive colonies per p60 mm. Alkaline phosphatase staining was performed using the Alkaline Phosphatase Detection Kit (Millipore) following the manufacturer’s instructions.

### Flow Cytometry

For assessing mitochondrial membrane potential and ROS levels by flow cytometry, cells, treated as indicated in the text, were trypsinised, resuspended in culture media containing 1% FBS and 100 nM TMRM (ThermoFisher Scientific), 50 nM MitoTracker green (ThermoFisher Scientific) or 5 μM MitoSox (ThermoFisher Scientific), and incubated at 37 ˚C in an incubator with CO_2_ supply for 10 min. Analytical flow cytometry measurements were taken using a FACSVerse flow cytometer (BD Biosciences) and analysed using FlowJo software (Tree Star Inc.). At least 10,000 events from each sample were recorded. TMRM and MitoSOX signals were relativized to MitoTracker green signal, as a proxy of mitochondrial mass.

### Western Blot

Cells transduced as indicated in the text were lysed on ice in RIPA buffer (50 mM Tris pH 7.5, 150 mM NaCl, 0.1% SDS, 1% Triton X-100, 0.5% sodium deoxycholate) supplemented with 100 mM NaF, 2 mM Na_3_VO_4_, 20 mM Na_4_P_2_0_7_, as protein phosphatase inhibitors, and 1 × complete protease inhibitor cocktail (Merck). Cellular lysates were used for immunoblotting with the indicated antibodies using standard procedures. Signals in western blots were detected using ECL prime (Amersham) and images automatically captured in an Alliance Mini HD9 (UVITEC) digital imaging system equipped with a 16-bit (65,536 grey levels) scientific-grade camera with variable electronic shutter speed and 4.8 OD dynamic range. Acquired images were processed using Adobe Photoshop CS6 and analysed with ImageJ software. The antibodies used were: rabbit anti-ACC 1:1000 (Cell Signaling, 3676), rabbit anti-FASN 1:1000 (Cell Signaling, 3180), rabbit anti-G6PD 1:1000 (Cell Signaling, 12,263), rabbit anti-UCP2 1:1000 (Cell Signaling, 89,326) and mouse anti-Tubulin 1:1000 (Santa Cruz Biotechnology, sc-32293).

### Glucose-6-phosphate Dehydrogenase (G6PD) Activity Assay

G6PD activity was determined by spectrophotometrically monitoring the increase of absorbance at 340 nm (A340) due to the reduction of NADP^+^ in the presence of glucose-6-phosphate. Briefly, cells were scraped and resuspended in ice-cold PBS containing 0.3% (v/v) Igepal 630 (Sigma-Aldrich) and 1X Complete protease inhibitor cocktail. Cell suspensions were sonicated three times for 30 s with 1 min rest intervals in a Bioruptor device (Diagenode) at 4ºC. The resulting lysates were cleared by centrifugation at 10,000 g at 4ºC for 10 min, and supernatants used for the assays. The protein content was measured with the Pierce BCA protein assay kit (ThermoFisher Scientific). G6PD reaction mixtures consisted of 30 mM Tris–HCl, pH 7.5, 6 mM MgCl_2_, 0.5 mM NADP + , 1 mM glucose-6-phosphate, and different amounts of cellular extracts in a final volume of 1 ml. Reactions were initiated by adding the cell extract to the reaction cuvette and analysed by recording A340 continuously in a UV 1800 spectrophotometer (Shimadzu) for 5 min at room temperature. Enzyme activity was calculated from the A340 slopes and expressed as nanomole of NADPH produced per minute and milligram of protein.

### Lipidomic and Fatty Acid Analyses

Liquid chromatography–mass spectrometry (LC–MS)-grade solvents and modifiers were: water, acetonitrile, and methanol (Fisher Scientific); and isopropanol, formic acid, ammonium formate and ammonium acetate (Sigma–Aldrich/Fluka).

Lipid standards LysoPC(17:0), PE(17:0/17:0), PG(17:0/17:0) and Cer(d18:1/17:0) were from Avanti Polar Lipids; carnitine(16:0) D3, PC(17:0/17:0), MG(17:0), DG(17:0/17:0), TG(17:0/17:0/17:0) and CE(17:0) were from Larodan; and myristic acid D27 from Sigma–Aldrich/Fluka.

Ultraperformance liquid chromatography (UPLC) separations were performed using an Agilent 1290 Infinity LC system (Agilent Technologies) with a pump (G4220A), a column oven (G1316C), and an autosampler (G4226A). Mass spectrometric (MS) detection was performed on an Agilent 6530 QTOFMS system (Agilent Technologies) equipped with an ESI source. For each polarity (i.e. ESI ( +) and ESI (-)), three functions were set-up. The first function collected the data without collision energy, while the second and the third function acquired the data with a collision energy of 25 and 40 eV, respectively (‘‘All Ions MS/MS’’). The acquisition rate was 8 spectra/s. The other parameters were MS1 mass range, m/z 50–1700; MS2 mass range, m/z 50–1700; capillary voltage, + 3 kV/-3 kV; nozzle voltage, + 1 kV/-1 kV; gas temperature, 325ºC; drying gas (nitrogen), 8 l/min; nebulizer gas (nitrogen), 35 psi; sheath gas temperature, 350ºC; sheath gas flow (nitrogen), 11 l/min. The instrument was tuned using an Agilent tune mix (mass resolving power 25,000 FWHM). A reference solution (m/z 121.0509, m/z 922.0098 in ESI ( +) and m/z 119.0360, m/z 980.0164 (acetate adduct) in ESI (-)) was used to correct small mass drifts during the acquisition.

Lipidomic analysis, cell lysis, protein precipitation, and total lipid extraction were performed by the addition of 300 µL of isopropanol (-20ºC) to 100 µL of cell suspension in PBS. 10 µL of a 50 µg/mL solution each one of myristic acid D27, carnitine(16:0) D3, LysoPC(17:0), PC(17:0/17:0), PE(17:0/17:0), PG(17:0/17:0), Cer(d18:1/17:0) MG(17:0), DG(17:0/17:0), TG(17:0/17:0/17:0) and CE(17:0) in isopropanol were added as internal standard to each tube. Samples were vortex mixed for 1 min. After 30 min of incubation at − 20 °C to improve protein precipitation, they were centrifuged at 16,000 g for 15 min. Supernatants were transferred to clean tubes and dried in a vacuum centrifuge. Samples were reconstituted in 100 μL of isopropanol/acetonitrile/water (2:1:1) (v:v:v) for its LC–MS analysis.

Lipids were then separated on an Acquity UPLC CSH C18 column (100 × 2.1 mm; 1.7 µm) (Waters). For analysis in ESI( +) mode the mobile phases consisted of (A) 10 mM ammonium formate in 60:40 (v/v) acetonitrile:water and (B) 10 mM ammonium formate in 90:10 (v/v) isopropanol:acetonitrile. The separation was conducted under the following gradient at a flow of 0.4 ml/min: 0 min 20% (B); 0–2 min 40% (B); 2–4 min 43% (B); 4–4.1 min 50% (B); 4.1–14 min 54% (B); 14–14.1 min 70% (B); 14.1–20 min 99% (B); 20–24 min 99% (B); 24–24.5 20% (B); 24.5–27 20% (B). Sample and column temperatures were maintained at 4 ºC and 65 ºC, respectively. The injection volume was 2 μl. For analysis in ESI(-) mode the mobile phases consisted of (A) 10 mM ammonium acetate in 60:40 (v/v) acetonitrile:water and (B) 10 mM ammonium acetate in 90:10 (v/v) isopropanol:acetonitrile. The separation was conducted under the following gradient at a flow of 0.6 ml/min: 0 min 15% (B); 0–2 min 30% (B); 2–2.5 min 48% (B); 2.5–11 min 82% (B); 11–11.5 min 99% (B); 11.5–14.5 min 99% (B); 14.5–15 min 15% (B); 15–18 min 15% (B). Sample and column temperatures were maintained at 4ºC and 65ºC, respectively. The injection volume was 5 μl. Lipid annotation was conducted using the R package LipidMS [36].

For fatty acid analysis from cellular lipids, cells were lysed by the addition of 100 µl of 0.1 M HCl in methanol (-20ºC) to 100 µl of cell suspension in PBS. 10 µl of a 25 µg/ml solution of PC (17:0/17:0) and myristic acid D27 in methanol were added as internal standard to each tube. Then, 400 µl of chloroform (-20ºC) were added to each tube, followed by vigorous vortexing for 1 min and centrifugation at 16,000 g for 5 min. The chloroform layer was then transferred to a glass tube, and the chloroform extraction step was repeated, followed by a combination of the organic layers and drying under nitrogen flow. Extracts were resuspended in 1 ml of 90/10 methanol/water containing 0.3 M KOH, and saponified in an 80ºC water bath for 1 h. After saponification, the samples were acidified by addition of 100 μl of formic acid, extracted with 1 ml of hexane (2X), dried under nitrogen flow, and resuspended in 100 μl of isopropanol/acetonitrile/water (2:1:1) (v:v:v) for its LC–MS analysis.

Fatty acids were separated on an Acquity UPLC CSH C18 column (100 × 2.1 mm; 1.7 µm) (Waters). The mobile phases consisted of (A) 10 mM ammonium acetate in 60:40 (v/v) acetonitrile:water and (B) 10 mM ammonium acetate in 90:10 (v/v) isopropanol:acetonitrile. The separation was conducted under the following gradient at a flow of 0.6 ml/min: 0 min 10% (B); 0–3 min 25% (B); 3–7 min 75% (B); 7–8 min 99% (B); 8–8.5 min 99% (B); 8.5–9 min 10% (B); 9–12 min 10% (B). Sample and column temperatures were maintained at 4ºC and 65ºC, respectively. A sample volume of 5 μl was used for the injection in ESI(-) mode.

### Respirometry

Basal and uncoupled oxygen consumption rates (OCR) and ATP production were measured using a Seahorse bioanalyzer (XF96 Seahorse Bioscience Inc.) and the Mito stress test kit (Seahorse Bioscience Inc). Cells (20,000 cells/well) were plated the day before the measurements on XF96 culture microplates (Seahorse Bioscience Inc.). Next day, the media was changed to XF Cell Mito Stress test pH 7.4 medium supplemented with 25 mM glucose, 1 mM Sodium Pyruvate, 2 mM Glutamine. The following inhibitors were used Oligomycin (1 μM), FCCP (1 μM), and Antimycin A/Rotenone (0.5 μM). Measurements were taken every 5 min after the addition of the drugs and results were normalized to the total cellular protein content determined by a BCA protein assay. Each experiment was conducted in triplicate and repeated at least 3 times.

### Statistics

Principal component analysis was performed on Pareto scaled and mean-centered data [37] using SIMCA-p + 12.0 PCA (Umetrics). The model quality was assessed by R2 (goodness of fit) and Q2 (goodness of prediction).

LC–MS data pre-processing was performed using MassHunter Workstation Sofware (Agilent Technologies). Hierarchical clustering analysis was performed using ClustVis platform.

Where indicated, Student’s t-test was used to estimate statistical significance between categories. Relative values (percentages) were normalized using arcsine transformation before carrying out their statistical comparison. Results are presented as mean ± SEM (standard error of the mean).

## Results

### Anabolic Metabolism is Activated by c-MYC Early in Cell Reprogramming

**Fig. 1:**
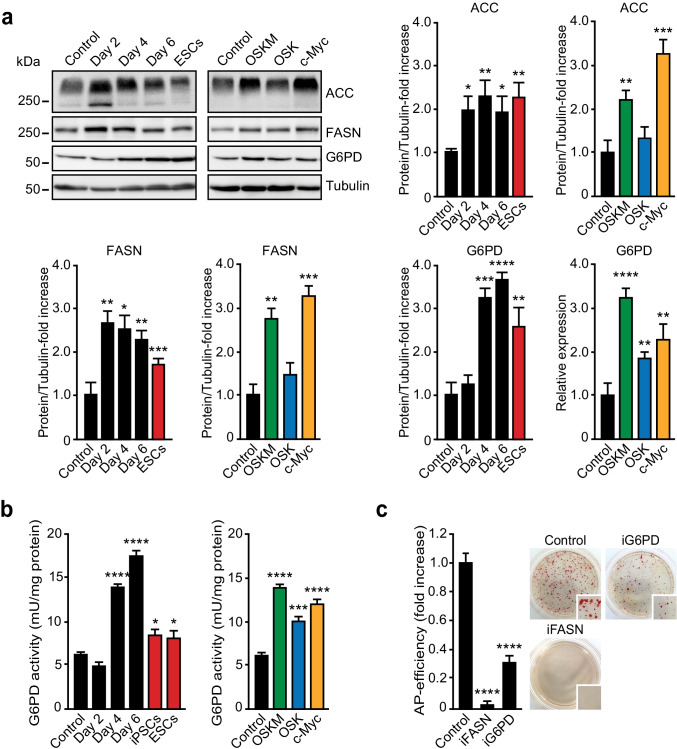
De novo lipid synthesis is induced by c-MYC. **a** Total cell extracts from ESCs, MEFs control, MEFs expressing OSKM for the specified days (panels on the left), or the indicated factors for 4 days (panels on the right), were analysed by immunoblotting using the antibodies shown. Graphs show the quantification of the indicated ratios (*n* = 3). **b** Bars chart showing the quantification of G6PD enzymatic activity in cellular extracts from MEFs transduced as in (a). **c** Bars chart showing the number of alkaline phosphatase (AP)-positive colonies obtained after 25 days of retroviral OSKM delivery and grown in the presence of DMSO (control), or inhibitors targeting FASN (iFASN) or G6PD (iG6PD) enzymes. Panels on the right, representative bright-field images from the plates of the indicated cultures after AP-staining. Inset shows a magnification of a selected area from the AP-stained plates. Statistics were from three independent experiments (*n* = 3), data are represented as mean ± SEM, one-tailed unpaired t-test, * *p* < 0.05; ** *p* < 0.01; *** *p* < 0.001; **** *p* < 0.0001

We and others have previously shown that, as in cancer cells [9], c-MYC induces a Warburg effect in cells undergoing reprogramming [6]. One of the metabolic hallmarks of proliferating cells is an increased of de novo fatty acid (FA) synthesis to fulfil the demand of building blocks for new membrane production. In agreement with their differences in proliferative rate [6], FASN and ACC levels were higher in pluripotent cells than in control mouse embryonic fibroblasts (MEFs), and the protein levels of these two genes increased during cell reprogramming in a time-dependent manner (Fig. 1a, upper panels). Relative to control MEFs, OSK-expressing cells (MEFs transduced with retroviruses encoding for OCT4, SOX2 and KLF4) showed similar levels of FASN and ACC at day 4 post-transduction. However, and relative to control cells, protein levels of both genes augmented in either OSKM- or c-MYC-transduced MEFs at day 4 post-transduction (Fig. 1a, lower panels). In addition to lipids, rapidly dividing cells have a large requirement for nucleotides and amino acids to sustain their high proliferative rate. In this regard, we observed increased G6PD levels in early cell reprogramming, pluripotent stem cells or in OSKM-, OSK- or c-MYC-expressing MEFs at day 4 post-transduction, relative to control cells (Fig. 1a). In agreement with the protein expression data, G6PD activity increased gradually early in cell reprogramming, and augmented twofold in OSK-expressing cells and threefold in either OSKM- or c-MYC-expressing cells at day 4 post-transduction (Fig. 1b). Interestingly, G6PD shunts glucose-6-phosphate, a glycolytic intermediary, to the pentose phosphate pathway (PPP) to produce ribose-5-phosphate, an important precursor for the biosynthesis of nucleotides, and NADPH, necessary to provide with reducing power in anabolic pathways [20]. In keeping with the results shown above, chemical inhibition of FASN or G6PD enzymes impaired cell reprogramming (Fig. 1c). These results suggest that de novo FA synthesis and shunting of metabolites from glycolysis towards PPP could represent valuable assets of cell reprogramming.

### Fatty Acid Composition is Remodelled in a c-MYC-Dependent Manner

**Fig. 2:**
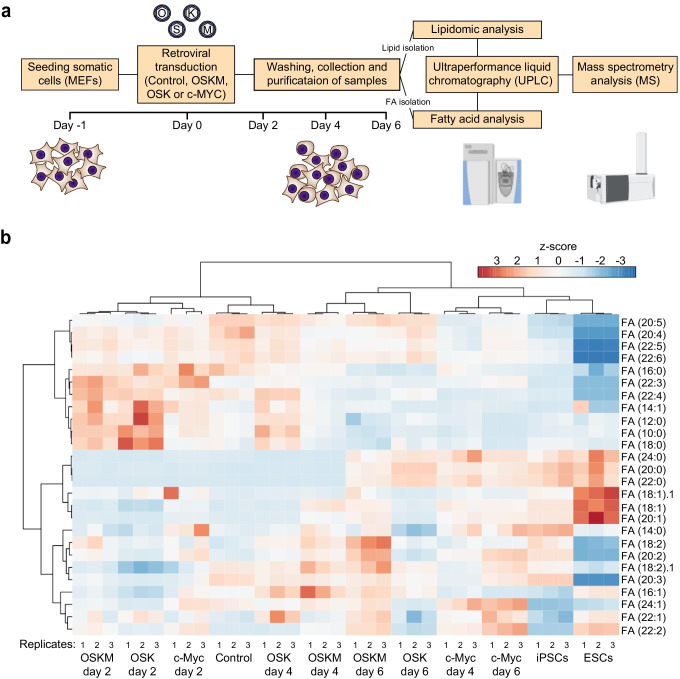
PSCs and MEFs display a different fatty acid profile. **a** Schematics depicting the workflow for lipid and FA profiling. Total lipid fraction was extracted from iPSCs, ESCs, MEFs control or expressing the indicated factors for the specified days was analysed by LC–MS to obtain the FA composition and lipid profile of the cells. **b** Heatmap illustrating the changes in fatty acid composition. FA species are indicated on the right as “carbon number(:)degree of unsaturation”. Heat colours represent z-scores of mass percentage of each fatty acid class (warm and cold colours depict higher and lower levels, respectively). Replicates are indicated at the bottom of the heatmap

**Fig. 3:**
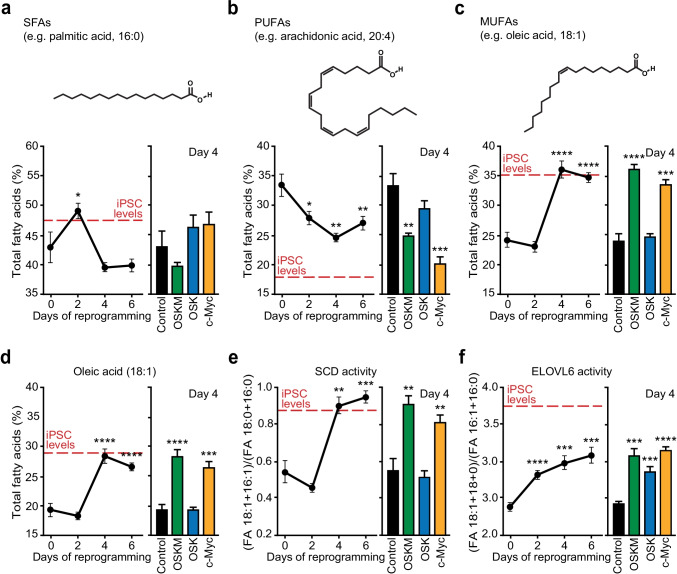
Fatty acid profiling during cell reprogramming is controlled by c-MYC. **a-d** Total lipid fraction was extracted from iPSCs, MEFs control or MEFs expressing OSKM for the specified days (graphs on the left), or the indicated factors for 4 days (bars chart on the right). Fatty acid composition was then analysed by LC–MS. Percentages of saturated (a), polyunsaturated (b) or monosaturated (c) FAs, and oleic acid (d) are shown. Chemical structures of palmitic acid (a), arachidonic acid (b) and oleic acid (c) are shown as examples of SFAs, PUFAs and MUFAs, respectively. **e** Estimation of stearoyl-CoA desaturase (SCD) enzymatic activity, measured as FA (18:1) + (16:1) to FA (18:0) + (16:0) ratio in lipid fractions, in pluripotent stem cells or MEFs transduced as above. **f** Estimation of elongase of very long-chain fatty acids 6 (ELOVL6) enzymatic activity, assessed as FA (18:1) + (18:0) to FA (16:1) + (16:0) ratio in lipid fractions, in pluripotent stem cells or MEFs transduced as above. Red-dashed lines represent FA levels and ratios found in iPSCs grown in 2i media. Statistics were from three independent experiments (*n* = 3), data are represented as mean ± SEM, one-tailed unpaired t-test, * *p* < 0.05; ** *p* < 0.01; *** *p* < 0.001; **** *p* < 0.0001

Our results showing the implication of *de novo* lipid synthesis early in cell reprogramming prompted us to investigate the variation of cellular complex lipid contents and the composition of their acyl chains in this process (Fig. 2a). Analysis of FA composition revealed profound changes in the relative amounts of different classes of FAs early in cell reprogramming (Fig. 2, 3).

Time-course experiments revealed significant differences in both length and saturation degree of the lipid acyl chains early during cell reprogramming. Compared to somatic cells, iPSC lipids displayed slightly higher relative levels of saturated FAs (SFAs) moieties (Fig. 2b), whereas iPSCs showed lower polyunsaturated FAs (PUFAs) and higher monounsaturated FAs (MUFAs) compared to control cells (Fig. 2b). In this regard, percentage of SFAs showed and initial peak at day 2 and plateau shortly after to the values displayed by MEFs at day 0 (Fig. 3a). We observed that the percentage of PUFAs decreased whereas MUFAs levels increased during the first stage of reprogramming in a c-MYC-dependent manner (Fig. 3b, c). In this regard, oleic acid (18:1) was found to be the most abundant FA induced by c-MYC during cell reprogramming, constituting the 30% of the total FA content at day 4 post-transduction (Fig. 3d). In agreement with the observed increase in MUFAs levels during cell reprogramming, the FA (18:1) + (16:1) to FA (18:0) + (16:0) ratio, which constitutes a reliable proxy for estimating stearoyl-CoA desaturase (SCD) activity, abruptly increased during cell reprogramming in a c-MYC-dependent manner (Fig. 3e), suggesting an activation of this enzyme by the proto-oncogene. In addition, we found an early and steady increase in the amount of 18-carbon acyl chains respect to that of 16-carbon FAs upon expression of reprogramming factors (Fig. 3f). As FA (18:1) + (18:0) to FA (16:1) + (16:0) ratio represents a good proxy for measuring elongase of very long-chain fatty acids 6 (ELOVL6) (and also FASN) activity, these results suggest that these enzymes are activated by the different reprogramming factors. Overall, these results support an active role for c-MYC in the remodelling of both length and saturation degree of acyl chains, as well as in the regulation of the enzymatic activities that control these processes early during cell reprogramming.

### Complex Lipid Diversity is Remodelled by c-MYC During Cell Reprogramming

**Fig. 4:**
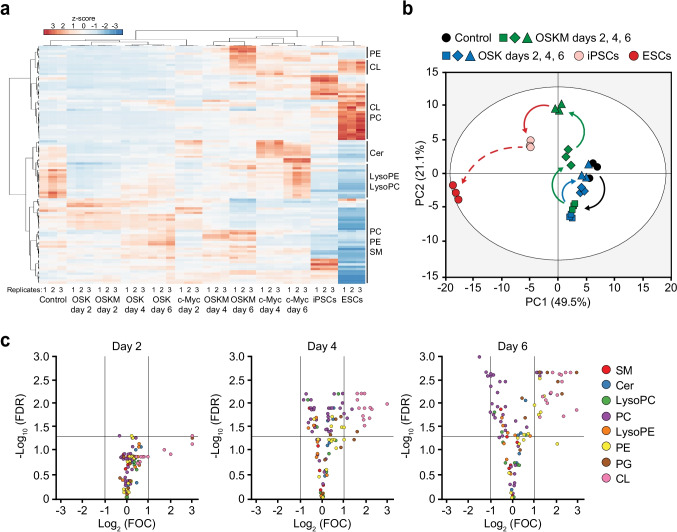
Remodelling of structural lipid composition during cell reprogramming is dependent on c-MYC. Total lipid fraction was extracted from iPSCs, ESCs, MEFs control or expressing the indicated factors, and lipids were then analysed by LC–MS. **a** Heatmap illustrating the changes in structural lipid composition upon OSKM, OSK, or c-MYC expression at the days indicated at the bottom. Lipid species are indicated on the right. Heat colours represent z-scores of mass percentage of each type of lipid (warm and cold colours depict higher and lower levels, respectively). Replicates are displayed at the bottom. **b** Principal component 1 and 2 (PC1, PC2) projections of structural lipid variations in OSKM- or OSK-transduced MEFs for the indicated days. Black arrow indicates the common transition observed in OSKM- or OSK-expressing cells for 2 days (green or blue squares, respectively). Blue arrow shows the reversal trajectory displayed by OSK-transduced MEFs at days 4 and 6 (blue diamonds and triangles, respectively). Green arrows display the observed transitions in OSKM-expressing cells at days 4 and 6 (green diamonds and triangles, respectively). Red arrow represents the hypothetic transition by OSKM-expressing cells to reach the lipid profiles found in iPSCs (pale pink circles). Red-dashed arrow illustrates the distance between iPSCs and ESCs grown in 2i (red circles). **c** Volcano plots represent the differences in structural lipid contents between OSK- and OSKM-expressing cells at the indicated days post-transduction. Left-upper or right-upper quadrants display the lipids upregulated unambiguously by OSK or OSKM, respectively. Cer, Ceramide; CL, Cardiolipin; FDR, false discovery rate; FOC, fold change; LysoPC, Lysophosphatidylcholine; LysoPE, Lysophosphatidylethanolamine; PC, Phosphatidyl-choline; PE, Phosphatidylethanolamine; PG, Phosphatidylglycerol; SM, Sphingomyelin

**Fig. 5:**
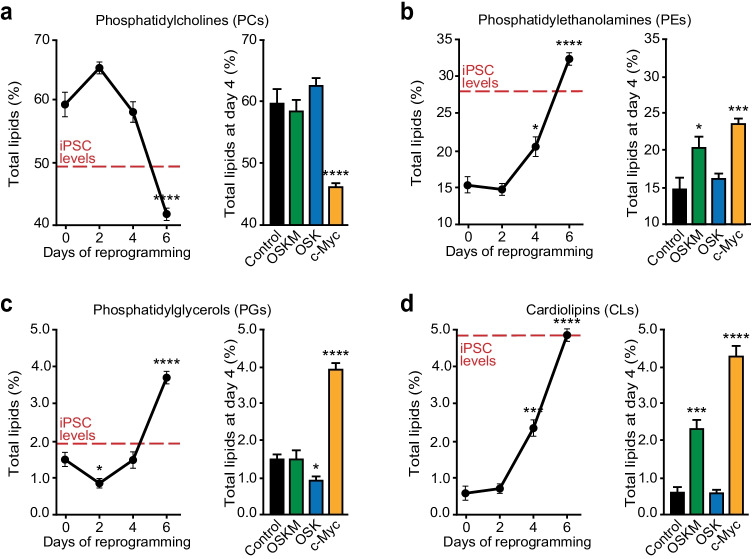
c-MYC expression induces upregulation of PEs, PGs and CLs and downregulation of PCs. **a-d** Total lipid fraction was extracted from iPSCs, MEFs control or MEFs expressing OSKM for the specified days (graphs on the left), or the indicated factors for 4 days (bar graph on the right) and analysed by LC–MS. Percentages of the indicated lipids are shown. Red-dashed lines represent the levels and ratios of the indicated lipids found in iPSCs grown in 2i media. Statistics were from three independent experiments (*n* = 3), data are represented as mean ± SEM, one-tailed unpaired t-test, * *p* < 0.05; ** *p* < 0.01; *** *p* < 0.001; **** *p* < 0.0001

In parallel to in FA length and saturation changes, lipidomics analysis revealed profound variations in the relative amounts of different classes of structural lipids early in cell reprogramming (Fig. 4, 5). While MEFs contained high relative levels of Lysophosphatidylcholine (LysoPC), Lysophosphatidylethanolamine (LysoPE), Sphingomyelin (SM), Phosphatidylcholine (PC) and Ceramide (Cer), pluripotent stem cells displayed low levels of these lipids and higher quantities of Cardiolipins (CL), Phosphatidylethanolamine (PE) and Phosphatidylglycerol (PG) (Fig. 4a).

Unsupervised hierarchical clustering showed that lipid profiles upon c-MYC overexpression grouped with those of OSKM-expressing cells (Fig. 4a). Interestingly, principal component analysis (PCA) of the lipidomics data revealed a common initial transition in both OSKM- and OSK-expressing cells (Fig. 4b, black lines). OSKM-expressing cells displayed a time-dependent connectivity in their structural lipid changes following day 4 post transduction, which may represent different lipid remodelling steps during their progression from the initial somatic state to pluripotency (Fig. 4b, green lines). Conversely, OSK-expressing cells reverted to their initial position after day 4 post-transduction (Fig. 4b, blue line), reflecting their lack of progression to pluripotency at these early stages. Solid- and dashed-red lines represent the distance between cells expressing OSKM for 6 days and iPSCs, and ESCs to iPSCs, respectively.

We next used volcano plots to compare the lipid profiles upon expression of OSKM or OSK at different days post-transduction. Relative to OSK-transduced cells, OSKM expression did not induce significant changes in their lipid contents at day 2 post-transduction (Fig. 4c, left panel). Interestingly, both combinations of reprogramming factors induced substantial changes in the cellular lipid composition as early as day 4 post-transduction (Fig. 4c, dots above the solid line). Relative to OSK-expressing cells, OSKM expression led to a specific upregulation of several CL species (Fig. 4c middle plot, upper-right quadrant). CLs representing the most significant changes in the cellular lipid contents found in OSKM-expressing cells at day 6 post-transduction (Fig. 4c, right plot, upper-right quadrant).

Time-course analysis of the changes shown by the specific structural lipids revealed that upregulation of PEs, PGs and CLs, and downregulation of PCs took place early in cell reprogramming, reaching the levels found in iPSCs at day 6 (Fig. 5a-d, graphs on the left). Also, these results revealed that the amounts of these lipids are highly influenced by c-MYC expression (Fig. 5a-d, bars diagrams on the right). Altogether, these results suggest that the diversity of structural lipids change early in cell reprogramming and that c-MYC plays a pivotal role in this remodelling along the course of the process. Yet again, our findings also illustrate that ectopic expression of OSK by itself does not constitute a compelling stimulus to induce the necessary changes for cell reprogramming to proceed.

### Cardiolipin Upregulation Correlates with Increased Mitochondrial Performance

**Fig. 6:**
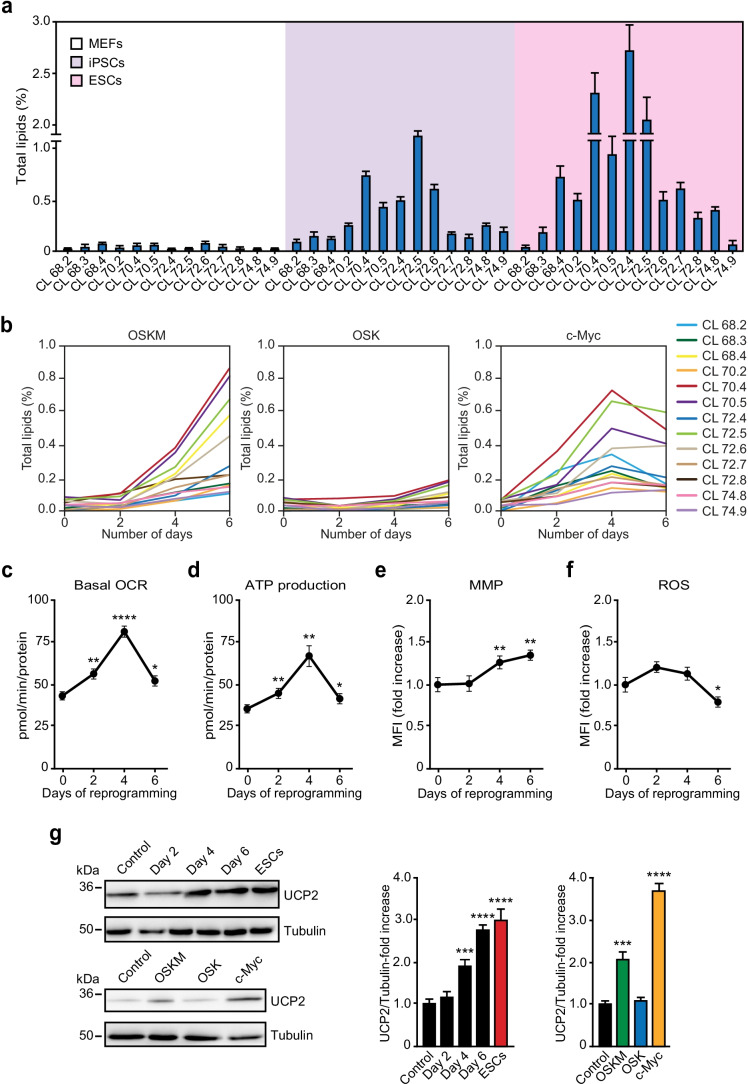
Upregulation of cardiolipins associates with improved mitochondrial performance. Total lipid fraction was extracted from MEFs, iPSCs or ESCs. Lipid composition was then analysed by LC–MS. **a** Bars diagram shows the levels of 13 different cardiolipins detected in MEFs (white background), iPSCs (violet background), or ESCs (pink background). **b** Percentage of 13 different cardiolipins (indicated on the right) detected in MEFs expressing the factors indicated at the top for the specified days (x-axis). **c-f** Basal oxygen consumption rate (OCR) (c), ATP production (d), MMP (calculated as TMRM to MitoTracker green ratio) (e), and ROS levels (calculated as MitoSOX to MitoTracker green ratio) (f) were assessed in MEFs expressing OSKM for the days shown in the x-axis. **g** Lysates from ESCs, MEFs control or expressing OSKM for the specified days (upper panels), or the indicated factors for 4 days (lower panels), were analysed by immunoblotting using the antibodies shown. Graphs on the right show the quantification of the data. Statistics were from three independent experiments (*n* = 3), data are represented as mean ± SEM, one-tailed unpaired t-test, * *p* < 0.05; ** *p* < 0.01; *** *p* < 0.001; **** *p* < 0.0001

Lipidomic analysis revealed that CLs were the lipid species that changed the most during cell reprogramming (Fig. 4, 5). Further analysis of lipidomic data identified 13 different CLs which, relative to MEFs, were abundant in both ESCs and iPSCs (Fig. 6a). The most represented CL in iPSCs were those with 70–72 carbons and 4–6 unsaturations in their acyl chains (Fig. 6a). Interestingly, a detailed analysis of changes in specific CLs during cell reprogramming revealed that the expression of OSKM or c-MYC alone increased their levels in a time-dependent manner (Fig. 6b). Conversely, OSK-transduced cells were unable to induce significant changes in CL levels during 6 days after expression (Fig. 6b). Altogether, these results suggest that CLs undergo a rapid increase during cell reprogramming in a c-MYC-dependent manner and that these mitochondrial-specific lipids may play an important role in pluripotent stem cells.

CLs have been described as mitochondrial-specific lipids that play a key role in shaping the structure and function of these organelles [38]. Interestingly, we and others have described an improvement in mitochondrial performance during cell reprogramming [6, 39–43]. In this regard, respirometry analysis of MEFs expressing OSKM showed that oxygen consumption rate (OCR) and ATP production were increased early in cell reprogramming (Fig. 6c, d). In parallel, analysis by flow cytometry revealed that MEFs transduced with OSKM gradually augmented the mitochondrial membrane potential (MMP) while reactive oxygen species (ROS) decreased during the same interval (Fig. 6e, f). Besides, we measured the levels of uncoupling protein 2 (UCP2), a main antioxidant enzyme in mitochondria. UCP2 protein levels were higher in ESCs when compared to control cells, and gradually augmented during cell reprogramming (Fig. 6g, upper panels) in a c-MYC-dependent manner (Fig. 6g, lower panels). Overall, these results show a correlation between the observed increase in CLs and the improvement of mitochondrial performance, suggesting that these mitochondrial-specific lipids may help to adjust mitochondrial activity while UCP2 may constitute a brake for the increase in ROS levels during ectopic expression of the reprogramming factors.

## Discussion

During somatic cell reprogramming cells undergo dramatic changes along their path to pluripotency, which include a profound reorganization of gene expression, energetics, cell–cell interactions, and lipid composition [4]. The results presented here reinforce the notion of exogenous c-MYC as the key factor orchestrating these changes. We found that, early in cell reprogramming, this protooncogene nourished biosynthetic metabolism by simultaneously increasing glycolytic flux, to increase the cellular concentration of glycolytic metabolites, and G6PD activity, to shunt these metabolites towards the pentose phosphate pathway and consequently feed anabolic pathways. The expression of c-MYC early in cell reprogramming stimulated lipid synthesis and outstandingly the remodelling of their acyl chains. Using lipidomics and liquid chromatography we profiled the profound transformation, dependent on c-MYC, of cellular lipid contents and their acyl chain compositions that are associated with cell reprogramming. Moreover, we found that mitochondrial fitness was improved in a c-MYC-dependent manner by increasing oxidative metabolism while increasing UCP2 amounts to keep ROS levels uphold; Thus, protecting cells undergoing reprogramming from the deleterious effects of these by-products of increased oxidative metabolism.

Cells need energy to guide non-spontaneous reactions and maintain cell homeostasis. In addition, proliferative cells have additional requirements for cell growth and division. However, some estimations suggest that the biosynthetic processes required to produce a new cell do not consume large amounts of ATP [44]. Conversely, they need to capture nutrients for producing biosynthetic precursors and coordinate the synthesis of macromolecules necessary for the generation of a new cell [20]. Cells with a high proliferation rate, such as PSCs or cancer cells, have a metabolism based on aerobic glycolysis, that is conversion of glucose to lactate to produce energy in the presence of oxygen [4]. This metabolic hallmark is known as Warburg effect. The high rate of aerobic glycolysis in these cells is critical both to produce ATP and provide intermediaries for biosynthetic pathways [45]. To ensure the availability of nucleotides, amino acids and lipids for cell proliferation, cells must provide a constant flux of the elementary building blocks for macromolecule assembly through these pathways [20]. Interestingly, it is broadly described that cells undergo a dramatic metabolic change during cell reprogramming, from an oxidative energetics in somatic cells, with elongated mitochondria, to a glycolytic-based metabolism in PSCs, with fragmented mitochondria [4]. In this regard, we and others have demonstrated that c-MYC increases glycolytic flux early in cell reprogramming [5, 6]. Now, our work suggests that in parallel to this metabolic conversion, pentose phosphate pathway, and de novo biosynthesis of fatty acids are activated early in cell reprogramming. Here, we demonstrate that c-MYC increases levels of G6PD, ACC, and FASN, which are key enzymes in these pathways, and that chemical inhibition of these enzymes dramatically impairs cell reprogramming progression [11]. In agreement with our results, several reports have shown how these enzymes play crucial roles in the maintenance of different stem cell populations [10–12, 46].

Exported citrate from mitochondria to cytoplasm is not only essential to provide acetyl-CoA for fatty acids synthesis, but also for protein acetylation. The high rate of citrate-derived acetyl-CoA production in primed ESCs is key for maintaining the histone acetylation pattern [47]. In agreement with data presented in this work, it has been shown that a decrease in the levels of cytosolic acetyl-CoA or an increase in lipid generation, as a result of the ACC activation, led to FIS1 stabilization and greater mitochondrial fission, thereby improving cell reprogramming efficiency [48]. Interestingly, we have previously described that c-MYC drives a DRP1-dependent mitochondrial fragmentation necessary for cell reprogramming [6, 34], suggesting that these processes may be finely coordinated during the stochastic phase of the process.

Our data also illustrates the remodelling of lipid contents during cell reprogramming, which affects the length and saturation degree of the acyl chains, as well as the total lipidome composition. Despite the extended list of cellular functions played by lipids, it remains a challenge for molecular biology experts to accurately explain the existence of such a broad diversity of lipid species in terms of specific cellular functions [49]. A satisfactory explanation for both chemical (lipid structures) and compositional (ratio of different lipids) extensive diversity of lipids in the cell may be the inherent consequence of the three properties of lipid enzymatic metabolism: 1) promiscuity, ability to recognize and combine a wide range of substrates; 2) preference, priority on one metabolite over others; and 3) redundancy, enzymes presenting overlapped functions. In summary, different levels of expression of redundant enzymes with different degree of preference and promiscuity may generate a broad spectrum of lipid compositions [49]. Here, we show that PSCs depict a completely different lipid profile than somatic cells: high contents in CLs and MUFAs, and low amounts of phosphatidylcholines and PUFAs. It has been described that the presence of a high proportion of PUFAs decreases membrane bending rigidity [28], an essential property to control cellular shape and endocytosis [29]. In this regard, dramatic cytoarchitectural changes take place during the stochastic phase of cell reprogramming [2]. Thus, a mesenchymal-to-epithelial transition occurs early in cell reprogramming [50, 51], showing downregulation of actin protein, a central player in defining cell shape and movement [52]. Our results, showing a c-MYC-dependent downregulation of PUFAs early in cell reprogramming, suggest that a high proportion of polyunsaturated acyl chains in cellular lipids is required to maintain mesenchymal-like morphologies in MEFs, rich in actin-dependent spikes and protrusions [53], but they become unnecessary once somatic cells become iPSCs, depicting a prototypical epithelial-like colony morphology, with tight aggregations of rounded cells [54].

Cell reprogramming and cell transformation show many similarities [4, 55]. Our results suggest that c-MYC induces upregulation of SCD enzymatic activity to increase MUFA levels early in cell reprogramming. Interestingly, it has been described that several cancer cell lines show high expression levels of stearoyl-CoA desaturase 1 (SCD-1), the most abundant desaturase enzyme, and that an abnormal FA composition, such as an increased MUFA to SFA ratio, is associated with an adverse prognosis in cancer patients [56]. In fact, some studies have demonstrated that SCD-1 and MUFAs play critical roles in the maintenance of stemness in cancer stem cells, becoming potential therapeutic targets in ovarian [57] or colon cancers [58]. The biological role of MUFAs in stemness remains elusive, however. It has been described that oleic acid induces non-canonical autophagy both *in vitro* and *in vivo* [59] and we found that oleic acid becomes the predominant FA in a c-MYC- dependent manner. Furthermore, a non-canonical autophagy pathway has been proposed to be necessary for cell reprogramming [60]. Thus, activation of non-canonical autophagy through some variants of MUFAs, such as oleic acid, may be necessary to guarantee a successful cell reprogramming process.

During the first days of cell reprogramming, a drastic increase in CLs biosynthesis was detected. CLs are essential structural lipids of the inner mitochondrial membrane that interact with a wide variety of mitochondrial membrane proteins to ensure the functionality of the electronic transport chain (ETC) complexes [61]. Specifically, CL interacts with ETC protein complexes and allows them to acquire their proper conformational structure for reaching their optimal enzymatic activity [62]. Specific binding sites for CL have been detected in complexes I, III, and IV [63]. Besides, to guarantee an efficient electron transport chain, ETC protein complexes are organized into supramolecular structures known as supercomplexes or respirasomes [64] and CLs are necessary to stablish this superstructure association [65]. In this regard, decreased levels of CLs affect the mitochondrial cristae structure [62], the assembly of respirasomes [63] and the macromolecular organization and function of ATP synthase [66]. Regardless of the metabolic switch observed during cell reprogramming towards a glycolytic-based energetics, OXPHOS is necessary early in this process [39, 67] and cells prone to reprogramming display a hybrid metabolism [6, 68]. In this regard, we observed that c-MYC-dependent CL upregulation correlates with the improvement of mitochondria performance. In agreement with our data, CLs enhance MMP, favouring proton pumping [69] and reducing ROS levels [38]. Interestingly, to maintain a proper cellular and genomic stability, ESCs express high levels of antioxidant enzymes, such as catalase, superoxide dismutase, glutathione peroxidase or UCP2 [70–72], which endows ESCs with the necessary molecular machinery to overcome the adverse effects of a ROS increase, such as oxidative stress-induced senescence [73]. In this regard, we found that UCP2 upregulation paralleled that of CLs in a c-MYC-dependent fashion. In this regard, it has been also described that CLs shape UCP2 structure and function through direct interaction with this mitochondrial uncoupling protein [74].

Overall, our work showing the early events driving the metabolic rewiring during cell reprogramming by c-MYC reinforce the existent parallels between cell reprogramming and tumorigenesis [4, 55]. Furthermore, and similar to the observed epigenetic conversion during cell reprogramming [75], our data also suggest that an analogous passive process may take place during lipid remodelling. Thus, once pluripotency-specific phospholipids begin to be synthesized in a greater proportion, somatic lipid species are diluted with each round of cell division. In this regard, the notion that targeted delivery of c-MYC-induced lipids to the correct subcellular compartment could facilitate the phenotypic transition to pluripotency of somatic cells deserves further investigation as it may bring about the modification of existent protocols to ease the process in the laboratory.

Finally, it does not escape to our notice that, during aging, mitochondrial function and CLs levels decrease while ROS and oxidized CLs accumulate [38]. Thus, it is not unreasonable to propose a role for c-MYC in restoring CLs and antioxidant protein levels, such as UCP2 [76], to guarantee an optimal renovation of mitochondrial function while ROS levels are upheld under control, reinforcing therefore the idea of cell rejuvenation early in cell reprogramming [40, 77].

## Data Availability

The data sets used and analyzed during the current study are available from the corresponding authors on reasonable request.
